# Medical Items

**Published:** 1898-07

**Authors:** 


### *MEDICAL ITEMS.*

---

Dr. J. L. Pope, of Athens, Ga., died of apoplexy June 9th.

---

Dr. V. H. Taliaferro has moved from Atlanta to Eatonton, Georgia.

---

The Medical College of Alabama opens its 33d session October 10. See their advertisement on page xxxii.

---

Dr. J. R. Reed, of Thomasville, Ga., retired from practice on account of old age and ill health, died June 12th.

---

The manufacturers of Mellin's Food have changed their name from The Doliber-Goodale Co. to the *Mellin's Food Co.* This is proper.

---

Dr. R. C. M. Page, so well known to many of our readers as Professor of General Medicine in the New York Polyclinic, died June 19th.

---

The Dios Chemical Co. has sent out a nice paper-weight of glass. Through the top is seen the names of Dios preparations. In the bottom is a mirror. Quite unique. Mailed free on request.

---

An Alabama woman has named her twins Fitzhugh Lee and Joseph Wheeler, but a Pennsylvania woman went her one better by naming her triplets Sampson, Schley and Dewey—and Dewey is a girl, too.—*Ex.*

---

We congratulate our friend and townsman, Dr. McRae, upon his selection for the address on surgery at the next meeting of the

American Medical Association. The choice was a good one, and Dr. McRae will demonstrate at the next meeting that the honor was worthily placed.

---

*The Philadelphia Medical Journal* offers $1,250 in prizes for the best monographs upon medicine, surgery, obstetrics, pathology or the specialties, the competition to close December 31st. Those wishing further information should address the Philadelphia Publishing Company, 1410 Chestnut Street, Philadelphia.

---

DO NOT put off sending bills quarterly, half-quarterly; you are giving your clients' warm gratitude too much time in which to cool. You yourself receive regularly every month your bills from the grocer, the tailor, the landlord, and are expected to quickly liquidate.—*Jour. Am. Med. Asso.*

---

WE desire to thank our friend Dr. H. F. Harris, now Associate Professor of Pathology in Jefferson Medical College, for interesting reprints upon "Amebic Dysentery." Dr. Harris's article in the *American Journal of Medical Sciences* for April upon this subject is a most valuable and complete paper.

---

THE surgeons who have thus far entered the volunteer service from Georgia are: Drs. Garrard, from Macon; Jarrell, from Savannah; Little, from Macon; Davis, Drake, Grandy, Moncrief, Geer, and Corput, from Atlanta; Harris, from Carrollton; Pate, from Unadilla; and Guinn, from Conyers.

---

IN case of fracture, non-union is frequently due to the presence of syphilis. It is well, as a matter of routine, to inquire as to the existence of this disease in any case of bone injury, since active anti-syphilitic treatment will greatly promote union and repair in any case in which the disease exists.—*Internat. Jour. of Surgery.*

EPILEPSY seems to be one of the chief infirmities of genius. Cæsar was an epileptic; Talleyrand is the authority for the statement that Napoleon had an epileptic fit at Strasburg just before the capitulation of the Austrian army; and Madden informs us that Byron had a number of slight attacks. Shakespeare makes frequent allusions to it, and Dickens gives a partial description of it in Walter Wilding.—*Medical Age.*

---

THE tenth volume of Transactions of the Southern Surgical and Gynecological Association is received. As usual, the Transactions contain a large number of interesting scientific papers upon surgery and gynecology by prominent Southern men. The next meeting of the association will be held in Memphis, the second Tuesday in November, 1898. The president is Dr. Richard Douglas of Nashville, and the secretary Dr. W. E. B. Davis of Birmingham.

---

AS TO articles in medical journals, every editor and experienced writer knows how much more acceptable, how much more likely to be read, are short, concise, rather than long and verbose ones. Every writer for journals should limit his article to an aspect as specific and single as possible, and not try to cover too much space or too many phases of a subject. Write more often if you please, but drive one nail at a time, and drive that home. It hardly needs saying that one should not attempt writing upon a subject until he is thoroughly certain he has something new or valuable to say. Writing for vanity's sake or to advertise the writer is the bane of medical literature.—*Philadelphia Med. Jour.*

---

THE American Pediatric Society is making a collective investigation of infantile scurvy as occurring in North America, and earnestly requests the cooperation of physicians, through their sending of reports of cases, whether these have already been published or not. No case will be used in such a way as to interfere with its subsequent publication by the observer. Blanks containing questions to be filled out will be furnished on application to any one of the committee. A final printed report of the investigation will be sent to those furnishing cases. Signed J. P. Crozer Griffith, M.D., *Chairman,* 123 South Eighteenth St., Philadelphia; William D. Booker, M.D., 853 Park Ave., Baltimore; Charles G. Jennings, M.D., 457 Jefferson Ave., Detroit; Augustus Caille, M.D., 753 Madison Ave., New York City; J. Lovett Morse, M.D., 317 Marlboro St., Boston, *Committee.*

---

FOLLOWING are the officers of the American Medical Association elected at the late meeting in Denver for the ensuing year: President, Joseph M. Matthews, Louisville, Ky.; First Vice-President, W. W. Keen, Philadelphia, Pa.; Second Vice-President, J. W. Graham, Denver, Col.; Third Vice-President, H. A. West, Galveston, Texas; Fourth Vice-President, J. E. Minney, Topeka, Kansas; Treasurer, Henry P. Newman, 100 Washington Street, Chicago, Ill.; Secretary, William B. Atkinson, Philadelphia, Pa.; Assistant Secretary, E. W. Woodruff, Columbus, Ohio; Librarian, George W. Webster, Chicago, Ill.; Chairman Committee of Arrangements, Starling Loving, Columbus, Ohio; Oration on Medicine, J. C. Wilson, Philadelphia, Pa.; Oration on Surgery, Floyd W. McRae, Atlanta, Ga.; Oration on State Medicine, Daniel R. Brower, Chicago, Ill. Place of Meeting, Columbus, Ohio, June 6–9, 1899.

---

THE FIRST NATHAN LEWIS HATFIELD PRIZE FOR ORIGINAL RESEARCH IN MEDICINE.—The College of Physicians of Philadelphia announces through its committee that the sum of five hundred dollars will be awarded to the author of the best essay in competition for the above prize. Subject: "A Pathological and Clinical Study of the Thymus Gland and its Relations." Essays must be submitted on or before January 1st, 1900. Each essay must be typewritten, designated by a motto or device, and accompanied by a sealed envelope bearing the same motto or device and containing the name and address of the author. No envelope will be opened except that which accompanies the successful essay. The committee reserves the right not to make an award if no essay submitted is considered worthy of the prize. The competition shall be open to members of the medical profession and men of science in the United States. The original of the successful essay shall become the property of the College of Physicians. The trustees shall have full control of the publication of the memorial essay. It shall be published in the transactions of the college, and also when expedient as a separate issue. Address, J. C. Wilson, M.D., 219 South Thirteenth Street, Philadelphia, Pa.

---

At a meeting held at the Academy of Medicine in New York City on the 24th of May, representatives from eight States were present to organize a "National Society for the Study of Epilepsy and the Care and Treatment of Epileptics."

Speeches favoring the formation of such a society were made by Dr. Abram Jacobi, Dr. Ira Van Gleson, Dr. C. A. Horter, Dr. Frederick Peterson, Dr. E. C. Fisher, and Dr. William P. Spratling, of New York; Dr. H. C. Rutter, of Ohio; Dr. William M. Bullard, of Massachusetts, and Dr. B. D. Evans, of New Jersey; and the following officers were elected: President, Hon. William Pryor Letchworth, LL.D., New York; First Vice-President, Dr. Frederick Peterson, New York; Second Vice-President, Professor William Osler, M.D., Maryland; Secretary, Dr. William P. Spratling, New York; Treasurer, Dr. H. C. Rutter, Ohio.

The society organized with forty-four members. Application for membership should be addressed to the Secretary at Craig Colony, Sonyea, N. Y.

---

BY some curious chance a copy of the late lamented *Magazine of Medicine* (once the *Moody*) found its way into the editorial rooms of the *British Medical Journal.* Ordinarily the editor of the *B. M. J.* is a very serious person, but that some things may move him to mirth is apparent from the following clipping from a late issue:

*The Magazine of Medicine*, a "medico-surgical literary journal," published at Atlanta, Ga., U.S A., is probably the only medical journal in the world which has a poet among the regular members of its editorial staff. Among the "associate editors" we note the name of "Wm. Colby Cooper, M.D., poet." If we were to confess that we had never heard the name of this medical bard before, we should doubtless have "Whom not to know argues oneself unknown" quoted against us; we therefore think it prudent, like Brer Rabbit, to "lie low and say nuffen." The poet can speak for himself. One day when the divine afflatus blew hot and strong upon him a strange thing happened—if we may be forgiven a phrase which overuse has made threadbare. He was carried away

On, on, out in planet-isled space, till at last
  A crystalline bound burst in sight,
With star-circled portal, high-vaulted and vast,
  All glowing with iridal light.

And I caught, in the instant the gates sprang ajar,
  The glimpse of a glittering throng,
And there fell on my hearing from hosts, near and far,
  The refrain of a triumphant song.

'Twill haunt me forever, through pleasure and pain,
  That song from Away and Above,
With its sweet and its solemn and sacred refrain:
  The Spirit of Beauty is Love.

We do not deny it. But as Mr. Weller, senior, said of matrimony, was it worth while to go through so much to learn so little?

We may inform our able English contemporary that although the *Magazine of Medicine* is now extinct, "William Colby Cooper, Poet, M.D.," still lives, in full possession of his poetic genius, and the "divine afflatus" still pursues him.

---

### "The Doctor."

BY C. E. L.

Oh, why not be a doctor, and for the public work;
Be vigilant and faithful, your duty never shirk?
There's lots of good that you can do among the common trash,
Who will always keep you busy if you do not mention—cash.

Don't be avaricious and wish that you were rich,
But labor for the people, who are wallowing in the ditch;
Sleep with one eye open, on dark and stormy nights,
For Johnny has the croup, and Jennie has no appetite.

What if you *do* go hungry, you're but a public dog;
You should wait upon all comers, in sunshine, rain, or fog.
Remember your profession is a very sacred thing;
Your duty is to cure men, not ask them for the "tin."

In cases of contagion, be never wanting there,
But hurry to the battle, though you yourself despair;
Don't run away from danger, but be always strictly "in it,"
And do your level best, though you lose your life next minute.

Should you by chance live many years, and follow my advice,
What multitudes of people will say that you were nice;
And when your labor's ended, resting in that narrow lot,
How many poor will bless you, and sigh for good old Doc.

—*Medical Age.*

